# New Developments in Transcriptomic Analysis of Synovial Tissue

**DOI:** 10.3389/fmed.2020.00021

**Published:** 2020-01-31

**Authors:** Hayley L. Carr, Jason D. Turner, Triin Major, Dagmar Scheel-Toellner, Andrew Filer

**Affiliations:** ^1^Institute for Inflammation and Ageing, University of Birmingham, Birmingham, United Kingdom; ^2^NIHR Birmingham Biomedical Research Centre, University Hospitals Birmingham NHS Foundation Trust, Birmingham, United Kingdom

**Keywords:** transcriptomics, synovium, sequencing, single-cell, microarray, stratification, fibroblast, pathotype

## Abstract

Transcriptomic technologies are constantly changing and improving, resulting in an ever increasing understanding of gene expression in health and disease. These technologies have been used to investigate the pathological changes occurring in the joints of rheumatoid arthritis patients, leading to discoveries of disease mechanisms, and novel potential therapeutic targets. Microarrays were initially used on both whole tissue and cell subsets to investigate research questions, with bulk RNA sequencing allowing for further elaboration of these findings. A key example is the classification of pathotypes in rheumatoid arthritis using RNA sequencing that had previously been discovered using microarray and histology. Single-cell sequencing has now delivered a step change in understanding of the diversity and function of subpopulations of cells, in particular synovial fibroblasts. Future technologies, such as high resolution spatial transcriptomics, will enable step changes integrating single cell transcriptomic and geographic data to provide an integrated understanding of synovial pathology.

## Introduction

Research into transcriptomic changes in the diseased synovium in rheumatoid arthritis (RA) has significantly enhanced our understanding of disease pathogenesis. This review details the research pathway taken to study gene expression in the synovium, including methods for synovial cell isolation, and analysis of tissue and cells by microarray and RNA-sequencing technologies. The progression of gene expression technologies which have increased our understanding of disease processes will be described, with a focus on understanding fibroblast populations, on which new transcriptomic technologies have had their most profound impact.

The synovium is a thin tissue lining the interior of the fibrous capsule, enclosing diarthroidal joints, and facilitating the normal function of the joint (1). In healthy, uninflamed joints the synovium produces compounds such as hyaluronate and lubricin. These are vital components of synovial fluid which fills the joint space and provides lubrication to aid load-bearing and flexion without damage to the cartilage and underlying bone (1, 2). By contrast, the inflamed synovium in RA is characterized by tissue hyperplasia and angiogenesis, accompanied by infiltration of multiple leukocyte populations, most significantly including monocytic cells, T and B lymphocytes. The latter frequently form organized aggregates that persist over time, releasing inflammatory cytokines and chemokines, and contributing to autoantibody production while remaining resistant to the normal processes of apoptosis and resolution that characterize acute inflammation (3, 4).

Fibroblasts play key roles within the RA synovium both in the regulation of leukocyte influx and efflux, and in damaging cartilage and driving indirect damage to bone (5–11). In RA, fibroblasts within the synovium become persistently activated, resulting in reduced responses to apoptotic signals, increased proliferation, production of proinflammatory molecules, such as IL-6 and CCL5, and the release of matrix remodeling enzymes, such as matrix metalloproteinase-3 (MMP-3) and MMP-9 (12–19). Consequently, the synovium develops an aggressive phenotype, leading to inflammation and hyperplasia, causing pain, and loss of function (17, 20). Whilst the role of fibroblasts in supporting these processes has been described *in vitro*, our knowledge of fibroblast heterogeneity and functions *in vivo* has been limited to decades-old histological observations of synovial microanatomy. This was dominated by the differing appearances of lining layer cells, which undergo hyperplasia in RA and appear to be continuous with destructive pannus tissue that damages cartilage, and sublining cells that are located alongside leukocyte infiltrates and synovial blood vessels. This heterogeneity led to synovial fibroblasts being termed fibroblast-like synoviocytes (FLS) historically, however recently developed transcriptomic technologies, specifically single-cell sequencing, have allowed for the recognition of subpopulations of fibroblasts. Understanding these subpopulations and their differing functions has the potential to open new doors for therapeutic intervention.

## Isolating Cells From Synovial Tissue

Studies are generally completed at two levels of resolution, from either whole-tissue or individual cellular populations. Whilst the methods for obtaining whole-tissue samples of synovium are fairly standardized (arthroplasty or biopsy), the methods of obtaining cellular populations are varied, which confounds the results of downstream analyses.

Two main methods, the enzymatic digest method and the explant-outgrowth method, are widely used to isolate and study synovial cells, in particular synovial fibroblasts. The enzymatic method of isolation focuses on disrupting the extracellular matrix and adhesion of cells to this matrix to create a suspension of synovial cells. However, a range of enzymes, concentrations, and lengths of digestion have been used to achieve this, with limited investigation of the efficacy of the isolation or the effect of the digestion on marker expression and cellular viability. This complicates interpretation when examining surface protein expression or gene expression *ex vivo*, making it challenging to compare between studies.

The explant outgrowth method is more consistent between publications, possibly due to the relative simplicity of the protocol. Small segments of synovial tissue are placed into tissue-culture, allowing adherent cells to migrate out of the tissue and begin proliferating (21, 22). After 7 days the remaining tissue is removed and the cells are cultured. Whilst easier to implement than the enzymatic digestion protocol, the explant-outgrowth technique is not without its caveats. There is a significant risk of selecting for synovial populations that proliferate rapidly and are able to migrate out of the tissue, meaning that other populations may not be accounted for.

To address the heterogeneity in isolation methods the National Institutes of Health (NIH) Accelerating Medicines Partnership (AMP, https://www.nih.gov/research-training/accelerating-medicines-partnership-amp), a consortium of research groups funded by government, industry, and non-profit organizations, developed optimized protocols for the isolation of cells from multiple tissues, including the synovium, alongside a protocol for the cryopreservation of synovial tissue allowing for later digestion, which aids in reducing batch effects (23). This approach brings consistency to the field of synovial dissociation, allowing for more robust comparisons between results from different research groups.

## Whole Tissue Approaches to Synovial Tissue Analysis

Studies investigating gene expression in the synovium initially took whole tissue approaches to assess broad, organ level changes. Whilst this work serves a purpose with regards to biomarker screening and understanding links between synovial and systemic inflammation, it is difficult to determine which specific cells are responsible for the changes in gene expression. Furthermore, subtle yet relevant alterations in gene expression amongst small cell populations are likely to be masked by changes in more dominant ones, meaning important mechanisms may be missed.

## Interrogating the Synovium by Gene Expression Array

Whilst the investigation of specific genes using PCR can be a powerful tool for testing pre-existing hypotheses, the ability to screen thousands of genes can extend gene expression analyses into discovery-focussed approaches. The development of cDNA microarray technology filled this niche. cDNA microarrays are inert supports, such as glass slides, upon which probes are printed or “grown” using masking techniques in spots, with each spot containing probes for a single gene (24, 25). In the most common platforms RNA is converted to cDNA before being labeled with a fluorescent dye. The cDNA is then hybridized to the probes in the microarray. After removing unbound RNA, the fluorescence measured from each spot can be used to calculate relative gene expression.

The strength of cDNA arrays is in the number of genes that can be simultaneously screened. Lindberg et al. (26) used an in-house generated microarray to investigate the expression of 16,164 genes in RA synovial tissue. The authors investigated differences in gene expression between synovial biopsy material obtained during arthroscopy and arthroplasty from several different sites within each joint to quantify the variation in gene expression dependent on the sampling procedure. Interestingly, even when non-inflamed biopsy samples were excluded from the analysis, a large number of differentially expressed genes were found between biopsy samples from the same joint. Despite this finding, the authors were able to identify patient-specific gene expression signatures, indicating that the variation imparted by the biopsy site does not completely obscure the variation between patients.

Several publications have investigated the contribution of anatomical origin to the variance seen in fibroblast gene expression. Chang et al. (27) and Rinn et al. (28) used microarrays to investigate the differences in gene expression between skin fibroblasts isolated from different locations of the body. The major determinant of differential expression between sites was shown to be amongst genes encoding proteins involved in cell development during embryogenesis. Fibroblasts isolated from the same anatomical location in different donors showed closer gene expression profiles than those from a different anatomical location in the same donor (29). This indicates that developmental pathways may impact more upon fibroblast gene expression than inter-individual variation, as might be expected for cells involved in tissue specific structural patterning.

Microarray platforms can be applied not only to whole tissue but to any sample from which sufficient RNA can be isolated. Microarrays have been used with laser capture microdissection, a technique for isolating specific regions of tissue from histological sections, to compare gene expression in the synovial lining layer between RA and osteoarthritis (OA) samples (30). One hundred ninety-seven genes were detected as differentially expressed between the two diseases. Samples clustered according to disease, indicating that the lining layer is significantly different in phenotype between RA and OA. Additionally, the RA samples could be sub-clustered into high- and low-inflammation samples, a feature that was supported by similar high and low levels of serum C-reactive protein or histological inflammation scores.

The concept of subclassifying RA samples on the basis of gene expression has been explored by other investigators. van der Pouw Kraan et al. (31) used hierarchical cluster analysis of microarray data generated from the synovial tissue of RA patients undergoing arthroplasty to find subgroups based on differences in synovial gene expression. In concordance with later work by Yoshida et al. (30), the samples clustered into high and low inflammatory groups. The high inflammatory group was characterized by high *CD3D, IL2RG*, and *CD8* expression, suggesting that these differences were driven by differential immune-cell infiltration into the synovium. However, the investigators subsequently showed that synovial groupings could be mapped onto the transcriptomes of synovial fibroblasts cultured from the same tissues, demonstrating a link between fibroblast, and leukocyte populations (32).

Microarrays can also be used to correlate gene expression with response to therapy. This holds particular importance as, without stratification, RA patient therapeutic responses are characteristically <70%, even to targeted biologic agents. If gene expression could inform which therapeutic regime is likely to have the most effect, it would not only benefit patients but also reduce the cost of care (33). One study used an in-house developed microarray targeting 17,972 genes to measure gene expression in the synovial tissue of 48 RA patients who responded to therapy to varying degrees, and 14 non-responders. The main source of variance in gene expression was the presence or absence of inflammatory aggregates within the synovial tissue, however no clear differences between responders or non-responders were found (34). In a seminal paper, Dennis et al. used microarrays to assess response to first anti-TNF therapy, discovering modules of response corresponding to dominance of different cell types (myeloid, fibroid, or lymphoid) that were correlated to immunohistochemistry (35).

Whilst the data provided by microarrays has provided insight into the pathogenesis of inflammatory synovial disease, this technology is limited in the number of genes that can be simultaneously assessed. With the development of RNA-sequencing that allows measurement of the entire transcriptome, attention has turned toward the use of these techniques and the unique opportunities, and problems, they present.

## RNA-Sequencing

RNA-sequencing (RNA-seq) offers significant advantages over microarrays with regards to assessing gene expression, namely not targeting a specific selection of genes. RNA-seq can measure protein coding and non-coding genes, and even micro-RNAs, increasing the utility of this tool. In addition, RNA-seq provides data over a larger dynamic range with lower background noise than microarray technology (36). Untargeted RNA-seq consists of the isolation of RNA and conversion to a cDNA library, which is then sequenced. Computational alignment to the genome or transcriptome can then be performed, avoiding the need for pre-selected targets.

Orange et al. (37) used bulk RNA-seq to investigate total synovial gene expression in tandem with histology, with an aim to subclassify RA. The samples could be clustered as high- or low-inflammatory tissues along with an additional mixed cluster. Six thousand five hundred eighty-two genes were detected as differentially expressed between the clusters, with the high inflammatory cluster expressing increased levels of genes associated with immune related pathways. Investigation of key histological variables in the tissue samples showed concordance with the sequencing results, with the high-inflammatory samples possessing high levels of immune cell infiltrate. Additionally, in a longitudinal consortium study taking synovial biopsies at first presentation of new RA, significant correlations were observed between histological type, whole tissue bulk RNAseq-derived gene clusters, and clinical response to first therapy (38, 39). This powerful approach elegantly demonstrates the predictive value of cellular gene clusters derived from whole tissue signatures.

## Single Cell Approaches to Synovial Tissue Analysis

**Figure 1:**
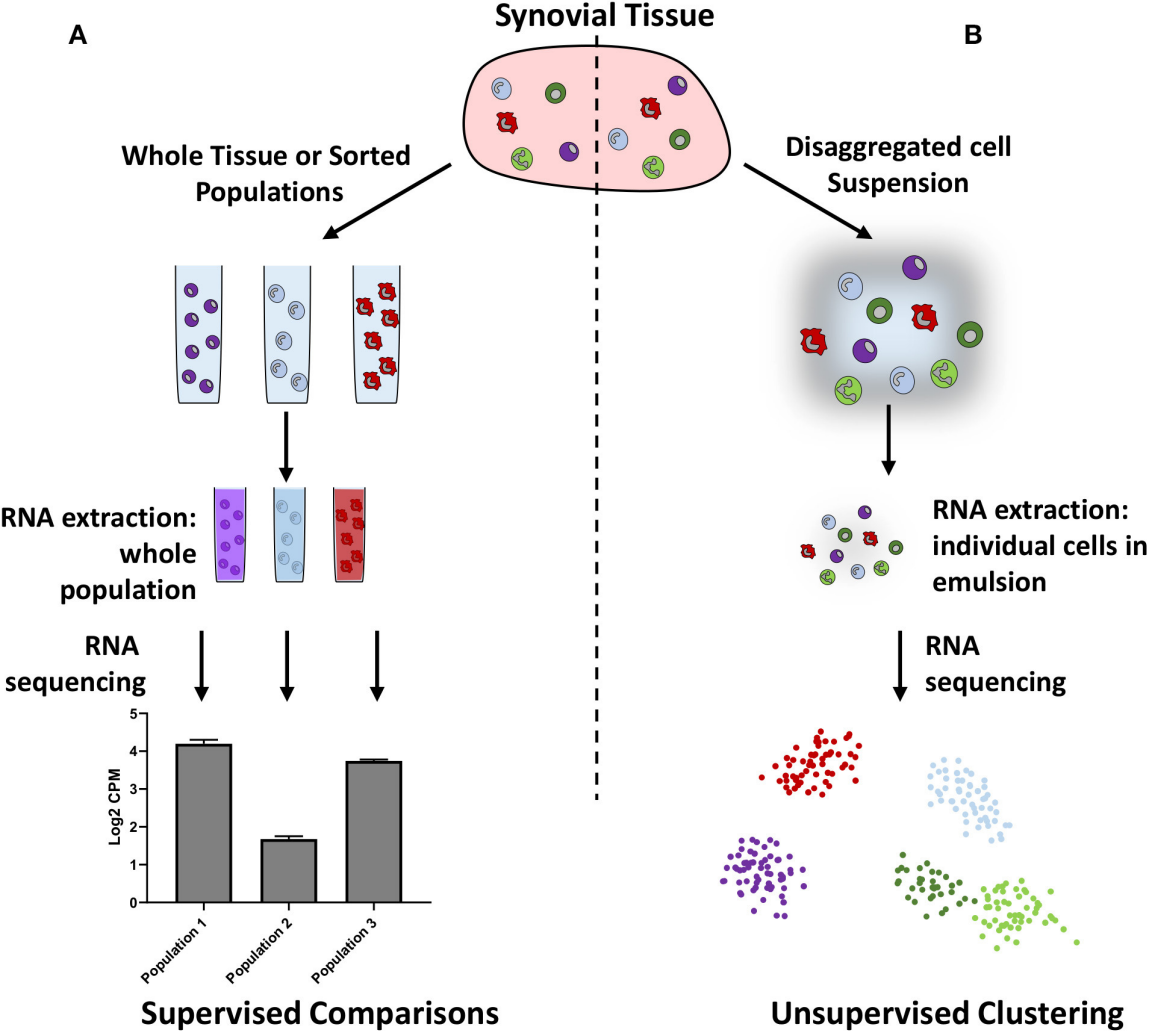
Schematic illustrating bulk **(A)** vs. single cell **(B)** RNA sequencing. Bulk RNA sequencing requires researchers to preselect cellular populations or sequence whole samples, whereas single cell RNA sequencing does not require pre-selection and can be used to identify cellular populations in an unbiased manner.

Although bulk RNA-seq provides in-depth information on gene expression in whole tissues or pre-defined populations of cells, assumptions made regarding the number and type of cells present may bias the results. Single cell RNA-sequencing (scRNA-seq) can reduce this bias by allowing independent sequencing of every cell within a tissue, or within a subset of cells (Figure 1). The subsequent use of unsupervised clustering techniques to find related cells, as defined by gene expression, can confirm the presence of previously defined populations or help in the identification of unknown populations of cells. However, caveats exist for readers of such papers: firstly, it is possible to over-cluster the data, leading researchers to believe there are more discrete populations present than actually exist. Secondly, multiple dimensionality reduction techniques and clustering methods exist, which can lead to different interpretations of the data, and which should therefore be clearly stated by authors and noted by readers. Thirdly, the depth of information obtained by scRNA-seq can be limited by low amounts of starting RNA, meaning that the absolute number of genes that can be recognized and quantified is lower than that of bulk RNA-seq. To leverage the strengths of this technique, both approaches are commonly used in tandem to identify key subpopulations and then further investigate gene expression.

Mizoguchi et al. (40) used a combined approach of microarrays, bulk, and single-cell RNA-sequencing to identify subsets of fibroblasts in synovial tissue from RA and OA patients. CD45^−^CD31^−^CD146^−^ cells were found to split into seven populations by flow cytometric analysis of CD34, CD90, PDPN, and CDH11 expression. However, following microarray and bulk RNA-seq of these populations, clustering of the data identified three populations in both datasets, CD34^−^CD90^−^, CD34^−^CD90^+^, and CD34^+^. scRNA-seq was then used on a small number of samples (2 RA and 2 OA) to confirm this finding. The CD34^−^CD90^+^ population was expanded in RA compared to OA samples, as measured by flow cytometry, possessed high *RANKL* and low *OPG* expression, and was capable of differentiating monocytes to osteoclasts *in vitro* suggesting a role in bone regulation and damage. The CD34^+^ population expressed high levels of *IL-6, CXCL12*, and *CCL2*, whereas the CD34^−^CD90^−^ population expressed high levels of *MMP1, MMP3, PRG4, HAS1*, and *CD55*. These findings indicate that the traditional split of lining vs. sublining does not capture the full heterogeneity of synovial fibroblast populations.

Unbiased single cell sequencing platforms have helped our understanding of the true heterogeneity of fibroblast subpopulations and therefore synovial pathology. Within the NIH AMP consortium, the exploration of synovial fibroblast subsets was extended through a combined investigation of larger numbers of RA and OA samples using bulk and scRNA-seq, and flow and mass-cytometry. Zhang et al. (41) used canonical correlation analysis to integrate this multimodal data from 51 RA and OA samples, facilitating a linked transcriptomic and proteomic analysis of single cells. In addition to defining the myeloid, T and B cell subpopulations present in the RA synovium, this study identified four synovial fibroblast populations: a CD55^+^ lining layer population and three sublining populations identified as CD34^+^, HLA^hi^, or DKK3^+^. With regards to gene expression, the sublining populations showed greater similarities with each other than with the lining population, a feature reflected in the shared enrichment for extracellular matrix related pathways within the sublining populations. HLA^hi^ synovial fibroblasts were the predominant source of IL-6. When classifying RA samples on the basis of immune infiltrate identified by histology, the HLA^hi^ population was expanded in leukocyte-rich synovium, hinting that this population may be driving classical inflammation within the synovium, whereas others, such as the CD55^+^ lining layer population, may be responsible for aspects of cartilage destruction.

The function of individual synovial fibroblast populations has been further explored using murine models of arthritis. Croft et al. (42) used the serum transfer induced arthritis model in combination with the deletion of fibroblast activation protein-α (FAPα) expressing cells to interrogate the role of synovial fibroblast subsets. FAPα is expressed on both lining and sublining fibroblasts in RA, therefore offering a mechanism for the global deletion of synovial fibroblasts (42–45). The deletion of FAPα^+^ cells during arthritis led to both a significant reduction in inflammation and accelerated resolution. This was accompanied by reductions in synovial cellularity, joint damage, and leukocyte infiltration, highlighting that the changes observed were not solely due to a reduction in the number of cells within the synovium. Bulk-sequencing of sorted populations mirrored the findings of Zhang et al. (41), with the largest differences being observed between the lining layer and sublining layer (45). Further similarities existed in the gene expression profile of these populations with the FAPα^+^CD90^+^ cells expressing higher levels of chemokines and cytokines, including IL-6, whereas the FAPα^+^CD90^−^ cells showed higher levels of RANKL and MMPs. The functional behavior of these populations was confirmed by adoptive transfer of the sorted populations into the joints of arthritic mice. FAPα^+^CD90^+^ cells increased inflammation but not joint damage, whereas the reverse was observed with the FAPα^+^CD90^−^ cells. Finally, scRNA-seq of murine synovial fibroblasts revealed that five independent populations could be found, one lining layer, three sublining, and an additional cycling population.

## Future Predictions

The investigation of synovial fibroblasts provides an excellent illustration of the power of single cell analyses. Our previous knowledge was restricted to speculation based on anatomical sub regions in the synovium and limited fibroblast surface markers. For the first time investigators have been able to identify and assign putative functions to individual fibroblast subpopulations that were previously unknown using the power of single cell sequencing. This provides exciting new opportunities to understand the pathobiology of inflammatory arthritis, and to target novel therapeutic approaches to fibroblast cells. Even the field of lymphocyte biology, in which multiple cellular subsets have already been identified, has been changed by single cell analyses of synovial tissue, as in the recent identification of a novel pro-inflammatory synovial T cell subpopulation in RA assisted by mass cytometry (46). Techniques such as cellular indexing of transcriptomes and epitopes by sequencing (CITE-seq) and RNA expression and protein sequencing assay (REAP-seq) are enabling the simultaneous resolution of transcriptomic and proteomic data at an individual cell level (47, 48).

The challenge now is to recast previously published findings into this new framework of individual subpopulations, and to integrate isolated cell transcriptomic and proteomic data with multiparameter platforms providing spatial proteomic and transcriptomic data alongside established techniques such as laser capture microdissection.

## Author Contributions

HC, JT, TM, DS-T and AF all contributed to the design of the review. All authors wrote, revised, and edited the final submission.

### Conflict of Interest

The authors declare that the research was conducted in the absence of any commercial or financial relationships that could be construed as a potential conflict of interest.
